# More harm than good? The questionable ethics of medical volunteering and international student placements

**DOI:** 10.1186/s40794-017-0048-y

**Published:** 2017-03-06

**Authors:** Irmgard Bauer

**Affiliations:** Division of Tropical Health and Medicine, College of Healthcare Sciences, James Cook University, Townsville, Qld 4811 Australia

**Keywords:** Humanitarian aid, Health care, Developing countries, Ethical concerns, Poverty, Exploitation, Social justice, Development industry

## Abstract

It has been argued that much of international medical volunteering is done for the wrong reasons, in that local people serve as a means to meet volunteers’ needs, or for the right reasons but ignorance and ill-preparedness harm the intended beneficiaries, often without volunteers’ grasp of the damage caused. The literature on ethical concerns in medical volunteering has grown tremendously over the last years highlighting the need for appropriate guidelines. These same concerns, however, and an appreciation of the reasons why current aid paradigms are flawed, can serve as indicators on how to change existing practices to ensure a better outcome for those who are in need of help. Such paradigm change envisages medical assistance in the spirit of solidarity, social justice, equality, and collegial collaboration.

## Introduction

Hearing from missionaries of the ‘physical misery of the natives in the jungle’([1], p.67), and guided by the parable of the rich man and Lazarus, the Alsatian pastor and organist Albert Schweitzer (1875–1965) wondered why rich European colonial nations did not do more to help the poor in Africa. Colonial doctors were employed to look after colonists and the troops, not to attend to local people. Schweitzer felt it was the Christian duty of a civilised society to send and support volunteering doctors in large numbers to ‘do good’ among the natives. Aged 30, he studied medicine and in 1913, with his wife - a nurse, founded the famous hospital in Lambarene/Gabon. His numerous communications, extended international fund-raising tours, and a Nobel Peace Prize in 1953 not only made him a celebrity but fuelled an enthusiasm in many at home to be part of such charitable work either as a volunteering doctor or nurse, or by fund-raising or knitting leprosy bandages. Schweitzer’s were not the only accounts of doctors’ services in the tropics, often romanticising notions of heroism, endurance and adventure. Times have changed. While *caritas* is still for many the reason to volunteer abroad, a long list of other motivations explains the enormous popularity of short-term medical missions and placements.

It is difficult to say when precisely secular medical volunteering started. From the 1960s, with the advent of government and non-government organisations (NGOs), and a lack of local qualified health staff in the ‘Third World’, doctors and nurses were sent on longer-term deployments applying western medicine in non-western environments. After the WHO Primary Health Care Conference in Alma Ata in 1978, the change in the prevailing concept of health care became evident in the literature though little related to medical missions. A further shift in world views in the 1980s questioned the morals of practices that do not consider their impact on others. The current grave ethical concerns about volunteering in general started towards the late 1990s, serious questions about the ethics of medical volunteering only rose over the past 10 years. This criticism may come as a surprise as it seems mean to question the noble task of helping the sick and injured in poor countries.

Matters came to light through observations in the field. For example, in 2000, a short paper appeared in the *BMJ* by two expatriate physicians working in a remote mountainous tourist area of Nepal [2]. In under a page, they brilliantly summarised their frustration at doctors and others turning up in the area ‘to do good’. With an existing local health system where locals’ skills were improving and confidence in the system growing, unsolicited ‘help’ was undermining all previous achievements. The main criticisms were: setting up ad hoc clinics along the trail – often near a local facility; ‘inappropriate arrogance’ assuming western medicine as superior; doctors working outside their scope of practice; ignorance of local language, culture and disease presentation; inappropriate treatment as ‘something must be given’; one-off consults with little follow-up and local staff having to deal with the consequences without patient records; and legal issues as doctors practised outside the national authoritative body. Concerns with ‘practising medicine on local residents’ related to dispensing of medication without records or consideration of local resistance, and the sale of adventure holidays for doctors where patients are recruited along the trail without connecting with the local health service and where equipment is brought in to treat chronic illnesses in one consultation.

Other blunt and unpalatable comments referred to self-serving neo-colonialism [3] or humanitarian neo-colonialism [4], the latter providing personal observations of the popular cleft lip/palate brigades where western doctors, contrary to the promised cooperation with local highly competent colleagues, performed most of the surgeries to train their own residents. In exchange for equipment, local doctors were forced to accept this ‘help’. That paper criticised the use of poor people in the Third World as ‘experimental fodder’ to improve one’s technical skills. Welling et al. [5] summarised their views, supported by vivid examples, as the ‘Seven Sins’, all of which will be covered in this article. Yet, not only qualified health professionals volunteer: the young ‘American on vacation’, proudly handing out to locals vitamins and antibiotics collected at home, a stethoscope around his neck conveying to the population a medical expertise that does not exist [6], or taking blood pressures [7]. An early analysis of two case studies in Latin America highlights the potential for insignificant or negative consequences of volunteering due to (then and today) absent systematic external evaluations [8].

Today, a voluminous body of literature exists on medical volunteering. Of particular value are articles that highlight concerns by providing numerous powerful examples from the field that reflect the complexity of the issues. This paper examines the ethical aspects in these recurring observations as they relate to medical volunteering, short-term medical missions and, due to the obvious parallels, to international student placements. But first, medical volunteering is positioned in the larger context of volunteering and voluntourism.

### The dark side of international volunteering

For a long time, tourism has been peddled to developing countries as the way out of poverty, creating health and well-being for all as a consequence, never mind that frequently the opposite is the case [9–11]. But there is a still better deal for disadvantaged countries: tourists who not only visit but volunteer their services. International volunteering thrives on the notion that global poverty and its consequences can be remedied by well-meaning people who even benefit from this exercise in various ways. Skills and experience are of secondary importance. A vast amount of literature criticises this activity as conveying a simplistic picture of development which re-enforces existing stereotypes and implicitly accepts structural inequalities [12–14]. Themes of neo-colonialism and exploitation recur, as do the many negative impacts, such as leaving locals with the product of pointless or questionable ‘work’, being a burden to the community, taking away local jobs, creating a dependency on foreign help, paralysing local initiative or ensuring that locals remain firmly at an assumed level of helplessness to secure more volunteer placements. Analyses by Guttentag [15, 16] are particularly useful in understanding the complexity of negative impacts. It was only a matter of time that the commercialisation of volunteering took a malevolent turn as exemplified in the much publicised criminal activities around orphanage tourism [17]. Finally, the excessive focus on the benefits for volunteers raises the question if volunteering for many is not simply a self-serving activity, redeeming oneself on a guilt-trip for the privileged middle-class [13]. As it turns out, the same concerns apply to medical volunteering.

### The many facets of medical volunteering

It is difficult to define a medical volunteer other than as a person with a qualification in a health profession who decides to offer these skills on a voluntary basis to residents of resource-poor regions. Apart from that, there is a wide range of variables, such as time commitments (from one day to several weeks), settings, the presence or not of a sending organisation, and any type of living and financial arrangements. Medical volunteers are not those with a salaried employment contract either with their own government, with the government of the receiving country or, for example, with the International Red Cross. To complicate things, there are also unqualified people, eager to do good or to enhance their holidays, and who are, unfortunately, accepted widely by commercial placement agencies, other sending organisations, or local facilities. A mission hospital reportedly recruited on the basis that all that was needed was the desire to help [7]. Health professionals have a range of options to volunteer their services. Many come across a local offer while travelling, others research for agencies, join a professional group (typically a specialist ‘brigade’ or a multidisciplinary team), or utilise links through a religious or educational affiliation. Two further opportunities to serve are through disaster aid or, as students or residents of health professions, via international training placements.

Disaster aid responds to an unexpected and immediate need for medical aid for large numbers of people due to natural disasters and is, therefore, in certain aspects different from mainstream medical volunteering. Numerous ‘lessons learned’ accounts [18–23] convey not only specialty-related experiences and harmful consequences of aid attempts [24], such as medical errors, the impossible follow-up of cases and staff distress. They also highlight repeatedly the importance of organisation, cooperation, and logistics, and the difficulties faced by the enormous influx of well-meaning unsolicited help hampering relief efforts. As a consequence, there are frequent calls for a much better preparation and preparedness in terms of global health training [25, 26] and within one’s profession [27, 28], as well as ongoing support pre, during and post deployment [29]. In a similar way, medical aid in war zones, though often longer term, also deals with large numbers of civilians and combatants in an unsafe environment and under challenging work conditions – probably better organised by selected organisations but staff are faced with similar issues [30].

International health training electives are increasingly incorporated into curricula, or provisions are made to use semester breaks for such placements with or without earning credits toward the qualification. Offered with a focus on under- or post-graduate students’ learning opportunities [31–33], it comes as no surprise that these placements are very popular in medicine, nursing and allied health professions with a particularly keen interest in surgical residencies [34–37]. However, strong ethical concerns have been raised [38, 39] identical to those discussed later on. In their particular role, ‘fearlessly confident’ [6] and overly enthusiastic, students are often culturally insensitive, disrupt the existing system with their mere presence, frequently are a risk to patients and to themselves, waste resources by religiously applying learned procedures inappropriately, and often are ‘student tourists’ with less time in the clinic and more in recreation [40, 41]. The preparation of a group of students going to the same locations may focus more on the trip itself rather than the learning experience [42]. Unfortunately, such electives can also have a less altruistic agenda, for example, where schools with large student numbers see overseas clinical places as mere opportunities to spread the load, or using such electives as a marketing tool to attract students. The placing of non-health and pre-medical students internationally is especially criticised, for its purpose is generally, and is marketed as such, to boost one’s CV and to be a more attractive candidate for medical school or other courses [43]. This does not preclude students’ eagerness to help but, unaware of the hidden cost and burden of their presence, they are given or take upon themselves medical tasks for which they are not qualified. This can easily foster in them the view that lower ethical standards are quite acceptable in a resource-poor setting [44].

Often overlooked is the role different sending organisations play in designing placement objectives and structure [45]. Academic institutions’ main interest lies in educational aspects, the sourcing and organising of placements, and the safety of students and mentors. However, their ethical responsibility goes far beyond to protect local communities, patients and colleagues from harm. NGOs work on the basis of their respective political or ideological objectives and need to examine carefully how these influence their approach to the provision of medical volunteering opportunities. Increasingly, NGOs are forced to commercialise in the highly competitive international volunteering market [46, 47] to the point, as one example from Guatemala shows, that an NGO which pays northern doctors US$ 500 per surgery is so popular that it needs to search the country for patients to meet the interest [48]. For-profit organisations, i.e. commercial placement agencies – regardless of their name, size and popularity – are businesses. To maximise profits, they must convey to the public a constant perceived need for outside help. The aim is to place as many volunteers as possible for as long as possible in the same location. Therefore, it is not in the agencies’ best interest to improve local health to the point that no volunteers can be placed and an income stream is cut off. Obviously, this contradicts their self-proclaimed purpose of eliminating poverty and assisting locals to improve health. The marketing line ‘over 20,000 happy volunteers’ [49] is not a recommendation but rather indicates the magnitude of the problem. Based on an outdated development paradigm, placements have now become ‘packages’ resembling mainstream mass tourism [47], even offering specials if booked before a specific time.

Finally, a brief comment regarding the site selection of placements. Oftentimes, the choice of a deserving community relies on personal or patronage relationships [48] rather than an actual need. The obvious clustering of volunteer positions in popular tourist areas testifies this. It would be hard for a poor community to reject a project [50], so the provider decides who is helped and where, typifying paternalistic health care [51].

### Ethical concerns in medical volunteering

The extraordinary complexity of ethical concerns in international volunteering applies in the same way to medical volunteering. Critical voices come from (ex-)volunteers, students on placements, local and expatriate health professionals, academics and patients. These concerns will be summarised here by addressing the context in which this work occurs, the shortcomings on the side of the ‘helpers’, and the issue of resources – guided, in part, by Wall’s structure of contextual features [52].

#### The context of structural violence

‘Structural violence is the combination of large-scale social, economic and environmental factors, incl. poverty, sexism, and political violence, that influence the poor health of people in developing countries’([52],p.81). Hence, short-term medical missions are, at best, a quick fix solution; at worst, they are perpetuating and supporting the factors that lead to poor health. Missions (surgical or otherwise) do not address health care problems, such as poverty and overstretched health care infrastructure [53, 54]. ‘Fistula tourism’ does not change a broken system [55]; without addressing a broken system, any ‘help’ can only be a short-term fix which may benefit individual patients but does not improve long-term access to quality health care. Many governments rely and depend on international volunteers, often with little to no regulation or coordination [51]. This dependence, and also the usually free volunteer services, may remove any incentive for a government to invest in health care or in preventative programs [53, 55].

Nowhere is this problem more evident than in the impact of western volunteers on local health services. Often, the western paradigm competes with rather than supports local health strategies [56]. The creation of duplicate or parallel health systems leads to an erosion of the local services with people rather waiting for the next arrival of free health care from overseas than consulting local personnel [8, 51, 53, 57, 58]. Over time, locals’ distrust leads to the services’ overall deterioration. Staff witness how patient numbers drop off when volunteers leave, modern treatment stops and drugs are running out [56]. Patients who can pay, but prefer to wait for free foreign help, are impacting on local health professionals’ earnings. Volunteers create local unemployment by substituting paid local colleagues with free alternatives [57, 59], pers obs IB]. Some local doctors and nurses, disheartened by their own and the local health system’s prospects, may choose to find employment overseas [60], leaving a hole that asks for even more volunteers. With most countries having their own (unemployed) medical and nursing graduates, offering free help may seem a cynical way of ‘development’, though most welcome by some governments’ budgets. Many volunteers may not be aware of this consequence.

#### The medical volunteers

##### Medical conditions

Depending on the location, medical conditions seen by volunteers are usually very different from those at home. Others may be the same but in a much more advanced state than ever seen at home, and risk/benefit of an intervention may be much harder to assess, for example, the increased risk of anaesthetics due to malnutrition or poor health [52]. The lack of diagnostic tools and resources, even of basics such as paper, equipment, medication, x-ray facilities or reliable power and water, limits the professional ability particularly of inexperienced volunteers to dangerous ‘help’ or at least questionable benefits for patients. Practising way beyond scope has been reported frequently; often, this is due to the absence of a more experienced colleague or because there is no one to whom to refer a patient, but it can also be in the keen and reckless pursuit of adventure. This leads to two serious problems: harmful treatment and the lowering of ethical standards.

##### Ethical double-standards

Throughout the literature, examples reveal a disregard of ethical codes, or an application of double standards [50], based on the fact that local patients have little or no choice but to accept the offered care. Therefore, a widespread view is that any care is better than nothing [7, 52, 61], even if it means dispensing useless tablets just because patients have walked far to the clinic – giving the volunteer a chance to look benevolent. The acceptance of lower standards in underprivileged settings (different countries – different rules) also absolve, of course, a volunteer of taking full responsibility for the delivered care [48]. What many volunteers do not realise is that they may work outside local legal governance and regulations [2, 6, 62], especially if they are working independently from an established organisation.

##### Harmful treatment

Numerous cases of harmful treatment have been reported. For example, based on an ill-informed declaration of lice as a health priority, insecticide-laced shampoo was dispensed to illiterate users for whom shampoo is a luxury to be shared with others [48]. Ignorance about cultural habits, living conditions or practical details reportedly harmed people, such as stomach ulcers by giving ibuprofen to people with limited water and food intake, reactions to antibiotics, or far-reaching and unexpected consequences of dispensing vitamins to children [6, 63]; bleeding after taking aspirin due to the risk of trauma in the countryside [24], or the provision of hip prostheses for people used to squatting [4], to list but a few. Medication labels in a language people do not speak where sharing is part of the culture [57], are an accident waiting to happen.

##### Volunteer needs prevail

Especially within the framework of training/learning overseas, interventions often take place for the volunteers’ own experience rather than a patient’s need [53]. ‘Trying out something’ on locals before using a technique at home [64], using local communities as a’practising ground for students’ [6] or using ‘poor kids’ for the training of western residents [4], presumably also to avoid costly litigation at home, have all been criticised profoundly. The question arises: training in precisely what? Are these skills then put to practice at home? Western health professionals needing to go overseas to learn something or get trained in particular skills only makes sense if there is an application at home.

##### Short time-frame

The intrinsic quality of short-term missions is precisely this limited time on location with considerable consequences to local health. The time is usually filled with the provision of treatments and surgeries, as many as possible to make the visit worthwhile. While volunteers can report how many patients they have seen in which timeframe (and secure the ‘bragging rights’ [5]), they have no way of knowing if the treatment was successful. Unless a condition can be treated completely in one visit, volunteers are unable to provide continuity of care, await lab results that may take longer than at home, deal with any complications on location and, overall, cannot be held accountable for their actions [52]. Often cited complications refer to post-cleft lip/palate surgeries with deaths due to underlying illnesses or excessive bleeding [5] though one must assume that those are more in the spotlight due to their popularity, and that many other interventions are simply not published to the same extent. The lack of follow-up through volunteers [53, 57] but also due to non-existing services also questions the wisdom of some interventions. For example, cleft lip/palate surgeries’ success is largely cosmetic; it is not followed up by speech therapy and other crucial long-term rehabilitation. An example of a double-amputee lady in a village without services, prostheses or rehabilitation [24], and presumably no prospect of making a living, highlights the often difficult decisions to treat or not to treat on the spot. Short stays have a particularly cruel psychological impact on vulnerable children in orphanages or health facilities as attachments are formed to the volunteers only to be abandoned again and again when the volunteers eventually leave [65].

##### Local health personnel

The short timeframe also impacts on local colleagues. The departing volunteers and teams leave the already overstretched local health service with the aftermath of all this help. After-care and complications are ‘dumped’ on local staff [66], often without being familiar with patients or their records. The additional workload also takes them away from their regular patients who may receive inferior care competing with staff time. Mopping up after the volunteers is not the only burden placed on local staff. Volunteers may expect them to arrange their travel, accommodation and touristic requests, especially difficult with larger visiting teams. Untrained, with limited qualifications, or highly trained, there appears a common impression by local staff of volunteers being insensitive, arrogant, disrespectful, undervaluing local knowledge and behaving superior [2, 8, 53, 55, 58]. Considering the lack of equipment, local health staff find the liberal use of disposable material upsetting, more so when they are reprimanded for wanting to re-use such precious commodities [67].

##### Cultural and language barriers

Encompassing the previous ethical aspects, problems occur due to ignorance of the local culture and at least some basic language [50], or due to superficial cultural assumptions [8, 68]. Humanitarian aid does not happen in isolation but within a multifaceted local health framework [69]. How patients understand the cause of illness (supernatural, microbes) influences how they comply with treatment plans [52]. The stigma of certain conditions needs to be understood. Poor communication and/or incorrect translation lead to incorrect diagnoses and inappropriate treatment [52]. The impatient disregard of culture and language problems, even if a patient’s confusion is obvious, is easily explained when the objective is to get a large number of patients ‘done’. Cultural disrespect is also displayed when volunteers turn up in shorts or ripped jeans where even the poor wear their ‘good’ clothes to a clinic [6].

#### The question of money

Much money changes hands in medical volunteering. Current figures on the cost of volunteering, i.e. fees, travel, accommodation, and donations, are hard to obtain as most calculations consider the economic value of volunteering as work donated. How much volunteers ultimately pay globally for the privilege of working overseas today is unclear. Around 10 years ago, the estimated value of the volunteering industry was up to £1.3 billion [70].

Depending on the arrangement, volunteers pay a hefty sum, often several thousands of dollars plus plane ticket and accommodation for a short stay on location. The average cost per volunteer 10 years ago, excluding travel and housing, was suggested at US$ 2400 [58], coinciding with the estimated $30,000 for a team of 10 volunteers which compared unfavourably with the $60,000 needed for a new 30-bed wing of the local hospital [55]. Elsewhere, the amount spent on T-shirts for the team would have funded the First Aid station for a year [57]. Volunteers themselves often question the use of their money when they feel taken advantage of, ‘overcharged’, and being part of a ‘cottage industry’; in a particular case, money went into the construction of new volunteer accommodation rather than a badly needed hospital wing, and the procuring of a constant stream of volunteers [7]. Sometimes, volunteers are only tolerated because their fees cover the entire budget of an organisation [59].

Apart from money, ethical concerns extend to donations of medications and equipment. The habit of donating to ‘get rid of unwanted stuff’ has been longstanding. From small-scale collections of personal medication left in a drawer to large-scale donations by companies of unused stock which can be written off against tax, the donors are usually more pleased than the recipients. Expired drugs, outdated textbooks or unsuitable equipment are offensive, can cause harm [41], and only shift the burden of disposal to those who can least afford it. Equally demeaning is the instruction not to touch the equipment left behind until the next set of volunteers arrives [56].

### Who benefits from medical volunteering?

The previous discussion raises the question whose needs such projects ultimately benefit [8]. The literature points firmly to the volunteers. Personal benefits and, therefore, motivations for volunteering, such as self-development, challenge, personal growth, feeling good about ‘giving back’, one’s standing among peers, CV enhancement, university credits, travel, and adventure permeate virtually the entire discourse on volunteerism (e.g. [71]). A more recent and highly concerning addition is volunteering as a requirement for university admission, hence forcing the participation in questionable and unethical activities. Presumably, the assumption is that volunteering demonstrates somehow a positive character trait of a person worthy of tertiary education.

The heading ‘How to feel really good after a vacation’ (‘doctors returning home rejuvenated’) [72] indicates another much-identified benefit, that one gained much more than one gave [73]. It has been suggested that international volunteering increases international awareness, social capital or career intentions [74], an enhanced understanding of cultural factors [75] and broader inequalities [63], but subjects in these studies were asked shortly after a placement; there is no evidence that any such benefit would last the time. In contrast, under unfavourable conditions, placements could increase prejudice and intolerance [76]. Medical experience, improved skills, career benefits [51, 77], the need for more satisfying work [78], the opportunity to try one’s hands on procedures not allowed at home [44] or to shine with heroic tales of actions or endurance [79] all prove irresistible magnets. The unbalanced focus on volunteers and their needs represents an egocentric approach to volunteering, ‘an outlet for self-centred interests and desires…’([80],p.98), ‘altruistic egoism’[51] or ‘… nothing more than a glorified form of tourism wrapped in a veneer of altruism with no sustainable benefit for the receiving communities’([81],p.4).

In contrast, there is limited evidence of long-term benefits to local communities, colleagues, or the health systems, not least because the questions of who decides what is a benefit, who gets it and how much is enough [63] have not yet been answered. There is no doubt that many thousand lives have been saved and patients’ health has been restored through immediate and appropriate interventions by medical volunteers. At the same time, developing countries have become the playground for some unwanted, inappropriate and harmful activities with minimal to no health improvement of the host communities. Structural violence, including poverty, corruption, and incongruent national priorities, provides the context in which local health services are struggling to provide care. It is tempting to suggest that low-income countries should just ‘do the right thing’ and focus less on armament and presidential mansions, and more on the well-being of their own people. However, the point has been made that waiting for this to happen only undervalues the current suffering of these same people [82]. Medical volunteering, as it happens today, has too many concerns attached, yet, these concerns are tangible. The question is how we can assist without leaving physical, mental and political debris behind.

### Potential approaches to a more responsible way of ‘helping’

#### Changing the approach

The current system of international aid is deeply flawed, its lasting benefits too often disappointing for two reasons ([83], p.135). First, currently, aid is all about delivering something from a provider to a recipient with the aim to fill gaps rather than build on existing capacities [83]. This method generates many of the problems already discussed which need addressing.

The often observed paternalistic approach where wealthy westerners decide what is good for poor locals and impose their own agendas, regardless of local assessments and priorities [50, 58, 59] needs to give way to responding in a collegial, collaborative and respectful manner. Schweitzer’s attitude towards Africans, now criticised as patronising and racist, reflects the *Zeitgeist* of his era; notions of professional superiority and arrogance have no place in the 21^st^ century. A volunteer who cannot see local colleagues and patients as equals should stay at home.

Most developing countries have their own medical and nursing schools. Rather than ‘helping tourists’, they need specialist training and continuing professional development. Continuous medical education (CME) is mandatory in western countries. Many volunteers report poorly trained health professionals and outdated methods. Education and skills transfer, including appropriate technology, are the one product that is badly needed [8, 53, 57, 62, 66, 67, 76]. A collaboration between professional associations to set up a country-appropriate long-term CME program appears more helpful than sending unskilled people. Local doctors, nurses and allied health professionals would feel connected and valued.

Second, standardised models, approaches or practices are applied in very different contexts and destinations. This is evident along the spectrum from short-term brigades to large international commercial agencies where anyone can volunteer anywhere. Short-term volunteering supports such one-size-fits-all practices. Individualised, needs-based collaboration takes more time and effort but promises better long-term sustainable results. The problems of short-term help can be alleviated through long-term relationships with local authorities to allow more structural, preventative policies and strategies to improve local health over time [50, 67, 84]. Sending organisations should strive for long-term partnerships, commit to repeated and predictable returns, and keep in contact [51].

#### Redirecting funds

Since poor health is predominantly a consequence of poverty, and the many millions spent in the medical volunteering industry seem, at times, grossly wasted, a more thoughtful use of the clearly available funds has been called for. The acute lack of resources on the ground suggests a wide range of opportunities for redirecting funds to where they are needed most, and where the well-known reasons for local poor health can be remedied without money being primarily used to finance volunteer experiences. Throughout the literature, the question has been asked if local services would not benefit more from the cost of plane ticket, accommodation and placement being donated [59] rather than from a questionable presence of volunteers.

Each situation and need is different but suggestions have been made, often by authors experienced in the field, on how resources might be able to do a better job. One option is to improve local working conditions [58] by contributing to local salaries [57]. Providing incentives to local health staff to work in underserved regions appears a better option than short-term volunteers who do not speak the language [57]. In the spirit of sharing, local health professionals could be invited to a tertiary care facility in their capital city, a neighbouring nation or a western country to update selected skills needed at home, and where different approaches to patient care, rather than inappropriate technology, can be demonstrated in daily work. For example, holistic care does not necessarily have to clash with the paternalistic understanding of care experienced in many poor countries. Donating CME or professional development courses, either in the country or abroad, would have far-reaching benefits.

Governments do have health plans but often not the resources for implementation [56]. Funds can be used to improve local services, or to provide financial support for patients who cannot pay [57]. More money could be spent on prevention so that, over time, fewer short-term volunteers are needed [57]. However, this may not sit well with some agencies or NGOs, not only for obvious commercial reasons. Repaired deformities provide photogenic marketing material; a patient rid of a belly full of worms not so much. Money is always needed for equipment and material. The supplies of hospitals, clinics or health posts can be replenished with donated funds, preferably locally purchased. Donations of medication and equipment should meet specific local needs, be mindful and be steered by the current WHO guidelines on donation of medicines, drugs, and equipment [85].

The reluctance to donate money to anonymous recipients is well-known. Money can go astray for many reasons and at many levels. This is upsetting even if it is understandable when some local staff may try to supplement their un-paid salaries. Rather than giving money to be used as needed, and to ensure that staff actually get what they need, it appears more helpful to direct donations towards a locally identified need. Collegial discussions direct how this need could best be met so that these items specifically are financed. Although collaborative ‘controlling’ of donations has been criticised as stifling the recipients’ freedom to manage the funds, one must also be mindful of the donors’ intended purpose of the gift. Overwhelmingly, intended local beneficiaries themselves would prefer more donor control over funds to ensure a fair distribution [83]. Monitoring the flow of funds is a delicate operation and can cause offense. There are many ways to procure what is needed transparently and without affront. In some situations it may be appropriate to pay/donate without cash changing hands. Mindful financial support does not remove a government’s obligation to pay its staff or provide resources to run a health service. One workable solution may be to agree that both sides contribute whereby any local contribution is matched by donors. The biggest challenge will be to identify projects and initiate the pathways for raising and distribution of funds. This, however, requires more commitment, energy, and insight than simply sending people abroad.

#### The importance of preparation

Preparation for overseas work is generally understood as pre-travel health advice. In contrast to regular travelers, international volunteers’ health risk profiles need to take into account additional aspects, such as different approaches to road traffic [86], altered living conditions, less ready access to medical care, and risks posed by their particular work. The need for appropriate pre-travel advice [87], medical and psychological assessments [88], and education and training in risk-reducing activities [89] has been alerted to. The importance of psychological support to avoid premature return has been recognised by Peace Corps from its inception [90]. Psychological distress is not restricted to work in extreme danger but has been linked to other problems, such as financial sacrifices, job insecurity or weak social networks, lasting months into the post-deployment phase. Careful pre-deployment screening and adequate measures to reduce the risk of mental illness are highly recommended [91]. The responsibility for the appropriate health preparation lies with the sending organisations which may employ in-house travel medical specialists or, like individual volunteers, consult a travel health professional or travel clinic. Apart from the personal misfortune, ill, injured or ill-adjusted volunteers add to the burden on the local system. Many short-term volunteers leave home with the basic ‘tourist health advice’. However, these preparations cover only one side of the coin, the well-being of the traveling volunteer. How is the volunteer prepared for the task ahead so that the local communities benefit from the often involuntarily received help?

Wanting to help is not enough. One must understand global poverty and its many derivatives, or medical help has a band-aid function at best. Prospective volunteers should educate themselves about the local social, political and economic conditions within which sit the local health realities. Such education is highly unlikely to come with ‘off the shelf’ projects for holidays. Volunteers should prepare themselves to make decisions without consultants, committees, and other staff. They should prepare for the experience, contact previous volunteers, learn about culture and language, and be aware of their own and any local limitations [52].

Much is written about the need to address the lack of responsible guidelines for clinical student placements as ‘we are moving away into a new era of global health medicine, away from the hero model that disempowers communities’([92],p.509). Students, residents, indeed anyone going abroad to train or learn must have adequate training that not only covers ethical issues, the causes of health disparities and anticipated cultural differences [81, 93] but also an understanding of vulnerable populations and, of course, solid technical skills. The overall framework should be one of transformative pedagogy [81] and social justice orientation [44]. To provide students with the opportunity to experience international health while minimising adverse consequences for locals [39], and to avoid the exploitation of one side for the benefit of the other, best practice guidelines have been suggested for sending and host institutions, trainees and sponsors [94]. Other examples are the ‘Ethical Challenges in Short-Term Global Health Training’ [95] based on case studies, though it is not clear if the cases were validated from a local perspective, and the ‘International Health Elective’ 4-week course [42] which includes (reciprocal?) peer-education. An increasing number of guidelines or preparation modules are being published more recently (e.g.[96]). All of them need to be subject to periodical examination for ethical appropriateness and timeliness.

Universities have a formidable responsibility in ensuring that placements do not use vulnerable people to practise clinical skills or teach inappropriate health promotion. Not only should there be no placement without thorough pre-departure education, such education should also modify any beliefs of offering something that is better than what locals can offer, or that a placement will truly change people’s lives; such attitudes can easily lead to disrespect and a sense of superiority [43]. If a preparation seems too much or too bothersome, students and universities need to ask themselves how serious they are about global health. It is the universities’ responsibility to ensure a comprehensive preparation. Universities should also select very carefully if they choose to utilise a commercial agency for their students to safeguard a seamless match between the preparation model and what this agency can offer.

#### Evaluating the outcome of medical volunteering on local health

Questionable practices and negative impacts of medical volunteering have been demonstrated at length. Such episodes, often brought to light by volunteers themselves, are unacceptable regardless of the potential outcome of the service and need to be addressed as a matter of urgency. This is independent of the need to demonstrate claims of benefits of voluntary medical care. In addition, nowhere else would it be possible to spend billions without justifying the expense to donors or sponsors. The outcome of poor practice can hardly be beneficial, but even ethically sound practices do not guarantee a positive long-term effect. Many have alluded to the pressing need for formal and systematic evaluations to validate assertions [8, 54, 57, 66, 97, 98]. Riding on the wave of charity and altruism, missions are disinclined to objective analysis [48], nor are teams typically equipped with skills to demonstrate impacts [54]. Rather, output in numbers of surgeries performed, patients seen, prescriptions filled, teeth pulled, or other services rendered [4, 6] justifies volunteers’ presence in resource-poor countries and pleases donors unaware that these numbers mean little in the overall context of a poverty-driven health status.

At first sight, two papers suggest a solution. One developed the ‘International Volunteer Impact Survey’ but the title is misleading as it focuses on the impact of service on volunteers [99]. The second promises a health impact assessment (HIA) tool for short-term medical missions but it is neither a HIA nor is it about impacts on locals [61]. Rather, it presents a tool to self-analyse a team’s quality of care. This is very useful and a good start but one must take care not to imply that, as a consequence of a positive self-analysis, the long-term impacts are positive as well. Unfortunately, no published application of this tool can be found.

In some countries, e.g. Guatemala [48], volunteer teams have been active for decades, yet, the countries’ or regions’ health status puts them at the bottom of their group. The obvious question is: Why? Long-term evidence of the outcome of services is needed to either continue with current practices, modify and improve, or terminate them. Genuine impact studies are highly complex and methodologically notoriously difficult. The ultimate need for measuring economic, political and health outcomes [53], and psychological, social and financial cost/benefits of volunteer work to verify claims [54] demonstrates this complexity. Throughout this paper, numerous ethical aspects have been identified, each able to serve in indicator-development or to raise research questions, such as: How many local health professionals have been crowded out of paid employment due to the free work of volunteers? How many local patients were deprived of care because staff had additional post-surgery patients to care for? How many patients did volunteers refer to local health services [55]? In a region with improving health status, how do we know this is due to volunteers? Could it not be a new road built to the community with subsequent improved nutrition? Could the area for some reason have become a government priority? Since local poverty is the reason for volunteer work, how did volunteers address poverty?

The need for continuing traditional epidemiological approaches to monitor local health status is self-evident, crucially in cooperation with the local authorities. However, the ‘real’ impacts of medical volunteering cannot be captured with population studies. They can only be demonstrated through local health professionals’ assessment of their (often involuntary) collaboration with westerners and through the experience of local vulnerable patients as recipients of that care. At this point, data collection tools designed around western aspects of interest become useless. To understand impacts, it is essential that outcomes are described based on what local patients and colleagues, not western eyes, see as important [83]. This requires a paradigm shift from ‘knowing what is best for you’ to being genuinely and selflessly interested in assisting people who need assistance. To obtain this knowledge, not only is a qualitative research approach essential (though only few examples exist so far [53, 62, 98]), but new outcome measures [98]. These indicators must originate in locals’ perception of health and well-being, and their need for assistance, to redress the current power imbalance between the decision-making giver and the powerless recipient [100]. An example of the creation of community-validated indicators has been described elsewhere [101]. To understand true outcomes, interviewing patients shortly after an intervention is in most cases not useful, but the difficulties in locating former patients, including the feasibility of travelling to their remote homes, pose challenges [54, 102]. As in western health contexts, many patients would not know if they have received substandard care; their gratefulness overrides any critical assessment. This is even more so in poor settings where patients have little choice but, with humbling trust in outsiders, accept whatever is given, or where culture or religion precludes any criticism of people in real or imagined authority. ‘Aid recipients have no choice but to praise, or the benefactor moves on’([59], p.231). A range of validated alternative research methods, such as visual or participatory approaches, would be highly appropriate to understand outcomes. It is important that the researcher is not identified as linked to the care givers by skin colour, language or origin. Information should be collected independently by locals who also have some insight into the complexity of a community and its former recipients of care so that the true effect of foreign medical interventions becomes visible. There is a moral obligation to not ignore local voices but make decisions together on how to proceed based on the findings. Otherwise, missions proliferate unregulated and unmonitored, and the lack of evidence only allows for more uncontrolled, self-serving exploitation of the poor.

### The way forward

Research in developing countries must follow stringent ethical guidelines. No such requirements exist for volunteering medical care; ‘charity’ seems good in itself [45, 63]. The following ethical principles have been suggested as a framework for monitoring medical volunteering: Establish a collaborative partnership (see also [103]), ensure fairness in site selection, commit to benefits of social value, educate the local community and team members, build the capacity of local infrastructure, evaluate outcomes, and engage in frequent ethical review before, during and after trips ([45], p.97–99). This scrutiny should apply to any aspect of medical volunteering but it highlights, first and foremost, the complex responsibility of the sending and receiving organisations which need to address and implement these principles. Ideally, individual travellers who happen upon volunteering opportunities should find themselves within such a regulated framework.

The onus of change lies 1) with the sending organisations irrespective of size or ideology, and 2) with the individual who wants to go overseas. Any sending organisation should demonstrate clearly the contemporary ethical underpinnings of its mission in practice and outcomes. Commercial placement agencies will have a hard time justifying their existence considering their business purpose. However, it seems that even some professional groups and university departments inexplicably choose to regress to times incompatible with current views on respect, dignity, equality, and human rights. Increasingly, the literature highlights the same issues again and again, yet, missions and student placements continue to grow exponentially with little concern for those aspects and, subsequently, for the people they are supposed to serve. Sending outside the overall context of solidarity renders the sending organisation (universities, NGOs, agencies) equally culpable of exploitation as the individual traveller searching for patients along the way. Consequently, ‘making a difference’ or ‘changing the world’ should not be used to advertise practices that do not even come close to this objective. Organisations are also tasked to select volunteers carefully who understand their work aligned with such concepts, not merely as charity [81] , clearly an ambitious challenge considering a volunteer’s often hidden personal motivations.

People who contemplate volunteering should carefully and honestly assess their motivations as well as their abilities and limitations. Many feel a genuine desire to help but, regrettably, such desire does not automatically translate into genuine help. Many who mean well will be unaware of the problems they may cause. At the other end of the spectrum, people volunteer in a reckless pursuit of their own interests using local people to achieve those goals. Many such volunteers have demonstrated that everybody would be better off had they stayed at home. Criticism of (medical) volunteering has long entered the social media informing prospective volunteers – and their parents – about the ethical and moral issues involved. Especially for young people it will be difficult to find a balance between the enthusiasm to go out and help the poor, and the decision to forego an opportunity when the set-up is clearly not in the local people’s interest. Ideally, one should strive to provide genuine assistance untainted by personal ambition or pecuniary advantages. Going abroad with a mental picture of how photos and stories will impress people at home starts already on the wrong foot. There are clear benefits in seeing the world; there is nothing wrong with learning something and getting experience. Volunteers should learn but, more important than any medical procedure is the understanding of the concepts of privilege, social inequality and political marginalisation so that they become world citizens with informed opinions who can use their voice against the drivers of perpetuated poverty and ill health.

## Conclusion

The aim of this paper was to present contemporary criticism of medical volunteering. A range of ethical concerns was identified and possible ways of alleviation suggested. Without a doubt, there may be many collaborations between western and local health professionals who work together in a mutually beneficial and respectful way to improve local health within the context of the local infrastructure. These may be at one end of the continuum of medical development aid, with unscrupulous commercial agencies at the other extreme. However, no individual and no project should be beyond scrutiny against moral and ethical requirements to demonstrate in practice and outcome that their presence helps improve local health. Poor local health professionals have no obligations to provide training experiences and practising arenas for western colleagues, neither do local patients.

As health professionals, we pledged ‘First Do No Harm’. This obligation has wide-reaching implications at any aspect of volunteering, starting by educating family, friends, colleagues, travellers and prospective volunteers who come for travel health advice. It also requires that we do more to validate outcomes of medical missions. Otherwise, not only time, energy, and money are wasted. Local disappointment, exploitation and putting personal agendas first can continue unchallenged. People who care enough about those in need will have no trouble going through the additional effort of ensuring that their activities genuinely improve lives; people who do not care should leave any development alone.

Considering the overwhelming impact of medical volunteering as it is practised today, should it not just be stopped? The clear answer is that millions of people depend on medical assistance. Stopping it would be distressing for both, the volunteers and the local recipients. However, continuing as is despite our insight into the potential harm is equally distressing. There is no recipe for how to get it right but the topics presented in this paper serve as a guide towards this goal. Rather than volunteering for earthly or heavenly rewards, selfless and genuine collaboration can improve people’s lives and bring about change, a change which perhaps needs to start with the ‘helpers’ first. In the end, after weighing up all the agendas, benefits and impacts on both sides, the balance must tip in favour of good local health.
